# The Magnitude of Optimal Antenatal Care Utilization and Its Associated Factors among Pregnant Women in South Gondar Zone, Northwest Ethiopia: A Cross-Sectional Study

**DOI:** 10.1155/2022/1415247

**Published:** 2022-09-02

**Authors:** Alebachew Taye Belay, Setegn Muche Fenta, Hailegebrael Birhan Biresaw, Yikeber Abebaw Moyehodie, Mequanint Melkam Yelam, Maru Mekie

**Affiliations:** ^1^Department of Statistics, College of Natural and Computational Science, Debre Tabor University, Ethiopia; ^2^Department of Midwifery, College of Health Sciences, Debre Tabor University, Debre Tabor, Ethiopia

## Abstract

**Background:**

Adequate antenatal care is essential for the health of the mother and the development of the fetus. The World Health Organization recommends at least four antenatal care (ANC) visits during pregnancy. In Ethiopia, only 32% of women of childbearing age attend four or more ANC visits. This figure is significantly lower than the average for least developed countries. This study is aimed at calculating the magnitude and identifying the factors associated with optimal antenatal care utilization in the South Gondar Zone, Northwest Ethiopia.

**Methods:**

A community-based cross-sectional study was conducted in the South Gondar Zone of Northwest Ethiopia from September 2020 to May 2021. A total of 434 participants were selected using multistage cluster sampling. Data were gathered through face-to-face interviews using a structured questionnaire. A multivariate binary logistic regression model was used to determine the factors associated with the optimal use of antenatal care.

**Result:**

The magnitude of optimal antenatal care utilization was 59% (95% CI; 54.20, 63.65). The study showed that mothers who completed their secondary school (AOR = 8.205; 95% CI: 3.406, 19.767), women who completed their tertiary school (AOR = 6.406; 95% CI: 2.229, 18.416), women whose husbands' level of education is secondary school (AOR = 5.967; 95% CI: 2.753, 12.936), those with a planned pregnancy (AOR = 1.912; 95% CI: 1.117, 3.271), those with a wanted pregnancy (AOR = 2.341; 95% CI: 1.366, 4.009), women whose husbands work in the government or nongovernment sector (AOR = 3.736; 95% CI: 2.093, 6.669), those not being exposed to the media (AOR = 0.520; 95% CI: 0.345, 0.783), and rural women (AOR = 0.267; 95% CI: 0.164, 0.435) were significantly associated with optimal ANC utilization.

**Conclusion:**

The findings suggest that more emphasis should be placed on education-based programs for women and their husbands that highlight the benefits of a planned pregnancy, desired pregnancy, and maternal health care. Meanwhile, the government and other concerned bodies should focus on expanding road accessibility, health institutions, and ambulance distribution to improve optimal ANC utilization in the area.

## 1. Introduction

The antenatal care (ANC) services provide an opportunity to prevent and manage existing and potential causes of maternal and newborn mortality and morbidity [1]. Antenatal care is supplied with the aid of talented healthcare providers to pregnant women and juvenile women to ensure the maximum extremely good well-being situations for each mother and baby throughout pregnancy [1]. In the past, the World Health Organization (WHO) has promoted 4 antenatal visits for single pregnancies. The first occurred in the 12 weeks following the age of gestation [2]. Antenatal care remains a major public health concern for the prevention of maternal and neonatal mortality worldwide by increasing the chances of access to vital obstetrics [3].

The high maternal mortality rate in certain parts of the world reflects unequal access to health services. Almost all causes of maternal mortality (99%) take place in the developing world. Over half of these deaths occur in sub-Saharan Africa and nearly one-third in South Asia [4–6].

The global reports in 2017 showed that only three in five women attended at least four antenatal visits. In regions with the highest rates of maternal mortality, such as sub-Saharan Africa, only 52% of women received at least four ANC visits [7]. The proportion of women with four or more ANC visits is considerably lower than the global average of 61.8% [2]. The prevalence of attending at least four or more ANC visits in 12 East African countries was 52.44% with the highest attending at least four or more ANC visits in Zimbabwe (75.72%) and the lowest attending at least four or more ANC visits in Ethiopia (31.82%) in 2012-2019 [2]. In the Ethiopian Demographic and Health Survey of women, 31.8% in 2016 [3, 8] and 43.11% in 2019 [4] received four or more checkups.

Most studies identified that the factors of antenatal care such as women's education [5, 6, 9], husband's occupation [6], socioeconomic status [5], place of residence [5, 10], understaffed health facilities [11], and distant ANC facilities [11, 12] were significantly associated with the use of antenatal care services.

The well-timed ANC usage can possibly save women from the direct causes of maternal and neonatal deaths associated with obstetric difficulty and might alleviate the consequences of pregnancy complications [13]. The enforcing and assuring usage of powerful maternity care of women in the growing international community is not a simple task [14–17]. In Ethiopia, the developments in maternal health care provide users with antenatal care, of which most of the women did not attend the minimal number of visits recommended by the World Health Organization [9, 18–20]. Most of the preceding research performed in Ethiopia meditated the low use of antenatal care in towns and cities [16, 19, 20]. Hence, this study is aimed at evaluating the coverage and risk factors of optimal antenatal care usage in the South Gondar Zone in Northwest Ethiopia.

## 2. Methodology

### 2.1. Study Setting, Data Source, and Study Design

This community-based cross-sectional study was conducted in the South Gondar Zone from September 2020 to May 2021. South Gondar is one of the zones found in the Amhara region and its capital city is Debre Tabor, which is located 666 km from Addis Ababa, the capital city of Ethiopia. According to the 2007 Ethiopian Central Statistical Agency Census, this area has a total population of 2,051,738, including 1,041,061 men and 1,010,677 women. Within this area, 468,238 households were enumerated, giving an average of 4.38 persons per household.

### 2.2. Population and Eligibility Criteria of the Study

All women aged 15 to 49 years who had at least one birth in the South Gondar Zone were considered source populations. All women in the reproductive age group who had at least one birth in the previous 1–2 years before this study were randomly selected, except the women who were not permanent residents in the study region.

### 2.3. Sample Size and Sampling

The two-stage random cluster sampling technique was used to select the sample. In the first step, a sample of three (3) districts, namely, Laygayint, Farta, and Fogera, was selected independently within the zone. In the second phase, a sample of four kebeles (enumeration areas) was selected independently in each selected district. Finally, the calculated sample size was proportionally allocated to the kebele group based on the identified number of eligible mothers. Mothers in each of the kebeles were randomly selected using a simple random sampling technique.

The sample size was calculated using single population proportion formula with the assumption: 95% confidence interval, 4.2% margin of error, 10% estimated nonresponse rate, and taking 23.13% proportion of optimal ANC utilization in Southern Ethiopia [21] then, the required sample size was 434.

### 2.4. Data Collection Procedures and Quality Control Measures

Data were collected using an interviewer-administered structured and pretested Amharic version of the questionnaire. The questionnaire was first developed in English and then translated to the Amharic language for appropriateness in approaching the study participants and then translated back to English by language experts to check its consistency. The questionnaire comprised sociodemographic, accessibility, behavioral, and obstetric variables. The training was given to data collectors and supervisors on the objective of the study, data collection procedures, data collecting tools, respondent's approach, data confidentiality, and respondents' right before the data collection date. The completeness of the questionnaire was checked every other day by the supervisors and investigators.

### 2.5. Variables of the Study

#### 2.5.1. Dependent Variable

The indicators that reflect maternal health services are antenatal visits during pregnancy (ANC). Therefore, the response variables are built from the questions included in the study questionnaire on antenatal care visits during pregnancy (ANC). The ANC was defined as adequate if the woman with a normal pregnancy had at least four antenatal visits and more than four in case of complications; otherwise, it was defined as inadequate.

#### 2.5.2. Explanatory Variable

The explanatory variable which may affect adequate antenatal care visits were age, religion, education level of women, occupation of women, education level of husband, husband's occupation, family size, monthly income, own mobile telephone, the decision of maternal care service utilization, access to mass media, residence, and marital status.

#### 2.5.3. Data Analysis Procedure

The data consistency and treating missing value exist in the dataset; data editing was conducted to check; data labeling, recoding, and exploratory analyses were performed using SPSS version 25 statistical software. The data were analyzed with statistical software R version 4.0.3. Descriptive statistics were used to submit data by the frequency with percentages in tabular form. A multivariate binary logistic regression model was used to determine factors associated with the optimum use of maternal health care. The result of the multivariable binary logistic regression model was reported in terms of the odds ratio adjusted to a 95% CI. All variables with a *p* value of 0.05 were found to be statistically significant.

#### 2.5.4. Ethics

The study focused specifically on women living in the South Gondar Zone that had at least one birth and aimed to get an insight into factors associated with optimal antenatal care utilization. Informed consent from the participants was acquired by the recruiting team. The recruiting team consisted of the caregivers to the women and the research assistants. Ethical approval was obtained from a research and Ethical Review Board committee of the College of Natural and Computational Sciences of Debre Tabor University, Ethiopia, with a code number of DTU/CNCS/855/21.

## 3. Results

### 3.1. Sociodemographic Characteristics of the Study Participants

A total of 434 mothers have participated in the study with a response. The average age of respondents was 33.63 years, with a standard variance of 7.19. About 188 (43.3%) of the respondents were in the age group of greater than 34 years. The majority of respondents 390 (89.9%) were Orthodox and married. Regarding educational status, 194 (44.7%) mothers and 155 (35.7%) respondents' husbands were unable to read and write. Regarding occupation, 157 (36.2%) mothers and 228 (52.5%) respondents' husbands were farmers. More than half, 252 (58.1%) of the respondents, were rural residents (Table 1).

### 3.2. Obstetric-Related Characteristics of the Study Participants

Approximately three-quarters, 321 (74%) of women, had their first pregnancy after the age of 19. Most, 369 (85.0%) of pregnancies, were wanted and planned. More than half of the 241 respondents (55.5%) had fewer than four children alive, while 193 (44.5%) reported having more than three children alive. About a quarter, 197 (45.4%) of the people interviewed, had their first pregnancies. Approximately 79 (18.2%) of the women had a history of abortion and sixty-eight (15.7%) of the respondents had a history of neonatal death. Nearly half, 205 (47.2%) of the respondents, were married between the age of 20 and 24 years (Table 2).

### 3.3. Prevalence of Maternal Health Care in South Gondar Zone

The prevalence of mothers' health care use is presented in Table 1. A total of 434 women participated in the study. The magnitude of ANC service utilization was 59% (95% CI; 54.20, 63.65) (Table 3).

### 3.4. Factors Associated with Four or More ANC Service Visits

The results of bivariate and multivariable binary logistic regression analyses were summarized in Table 4. The model revealed that maternal education, maternal occupation, husband's education, husband's occupation, media exposure, time taken to get the nearest health facility, planned pregnancy, wanted pregnancy, health profession preferred by women, source of information about maternal care, media exposure, place of residence, residence, duty service of maternal health care, number of living children, and road accessibility to the nearest health facility, health provider behavior, presence of health facility in kebele, transportation, and family size were significantly associated with four or more antenatal care visits.

The results of this study showed that education had a positive association with 4 and more ANC service uptake, meaning that the amount of ANC service uptake increased as the woman's and husband's education levels increased. Women who had primary educational status were 12.431 (AOR = 12.431; 95% CI: 5.739, 26.929) times more likely to receive four and more antenatal care visits when compared to illiterate women. Women who have attended secondary and tertiary level education were 8.205 (AOR = 8.205; 95% CI: 3.406, 19.767) and 6.406 (AOR = 6.406; 95% CI: 2.229, 18.416) times more likely to receive four and more antenatal care as compared to illiterate women, respectively.

Women who had planned and wanted pregnancies were more likely to use four or more ANC services than women who had unplanned or unwanted pregnancies. When compared to women who had an unplanned pregnancy, women who had a planned pregnancy were 1.912 (AOR = 1.912; 95% CI: 1.117, 3.271) times more likely to use four or more prenatal visits. Additionally, When compared to pregnant women who had an unwanted pregnancy, those who had a wanted pregnancy were 2.341 (AOR = 2.341; 95% CI: 1.366, 4.009) times more likely to use four or more prenatal care visits.

Four or more ANC service uptakes for husbands with primary education were 2.309 (*AOR* = 2.309; 95% CI: 1.272, 4.193) times higher compared with illiterate husbands. Similarly, four and above ANC service uptakes for husbands with secondary education were 5.967 (*AOR* = 5.967; 95% CI: 2.753, 12.936) times higher compared with illiterate husbands. Women who lived in urban areas were more likely to use four or more ANC services than women who lived in rural areas. Four and above ANC service uptakes of the rural women were 0.267 (AOR = 0.267; 95% CI: 0.164, 0.435) times lower compared with urban women.

Women whose husbands worked for the government or nongovernment sector were 3.736 (AOR = 3.736; 95% CI: 2.093, 6.669) times more likely to receive four or more ANC service uptakes than women whose husbands worked as farmers. Media exposure was linked to the use of four or more ANC services. Women who were exposed to the media were more likely to use four and more ANC services than women who were not exposed to the media. Women who did not have access to the media were 0.520 (AOR = 0.520; 95% CI: 0.345, 0.783) times less likely to attend four ANCs than those who did. Finally, women who had to travel more than two hours to reach the nearest health facility were 0.332 (*AOR* = 0.332; 95 percent CI: 0.146, 0.757) less likely to receive four or more ANC services than their counterparts (Table 3).

## 4. Discussion

This study assessed the risk factors of coverage and associated risk factors of optimal antenatal care utilization in the South Gondar Zone. The study illustrated that the proportion of optimal antenatal care utilization was found to be 59.0%. This antenatal care utilization is lower than 60.2% in the Indonesia Demographic and Health Survey 2017 [22]. However, this antenatal care utilization is higher than 30% in the Bangladesh Demographic and Health Survey 2014 [23], 58.53% in Sub-Saharan Africa from 2006 to 2018 [24], 35.3% in Debre Tabor Town, Northwest Ethiopia, 2017 [25], 46% in Rwanda, 2014 [26], 58% in Nigeria using the 2018 Nigeria Demographic Health Survey [27], and 52% in Tharaka Nithi County, Kenya, 2016 [28].

The educational level of women was the factor to receive four and above antenatal care visits. This finding was consistent with the study conducted in Amhara regional states, Ethiopia [29], Ethiopia [30], and Vietnam [31]. It showed that the increased educational level increases women's received optimal ANC visits. Furthermore, the husband's education is also a factor of women who use ANC visits. Similar findings were reported in studies conducted in Ethiopia [32]. Most women in the South Gondar Zone are socioeconomically dependent on male partners who make household decisions, and this has an impact on maternal health care utilization [32]. It is because educated mothers are thought to be more aware of the importance and availability of ANC services and more likely to use them. Education provides women with the opportunity to be empowered for more confident and capable decisions to use modern health care services for themselves and their children. Furthermore, it increases women's knowledge on where and how the best health care can be accessed and enhances women's capability of making autonomous decisions [33].

Women who had planned and wanted pregnancies were more likely to use four or more ANC services compared to women who had unplanned or unintended pregnancies. This finding is consistent with studies done in East African Countries [34]. This may be because mothers who plan to have a child want to have a safe pregnancy and therefore pay special attention to their prenatal care. Additionally, the wanted pregnancy of women has increased the use of ANC visits. This finding is consistent with studies done in Ethiopia. This could be because mothers who want to have a child want a healthy pregnancy and hence pay special attention to their antenatal care.

Place of residence was a factor of women to use four or more ANC services. This result was consistent with those of studies in Ethiopia [25]. Women who live in an urban area may have easier access to material resources (such as money, automobiles, or motorcycles) that can make ANC services more accessible. This could be attributed to the fact that urban women are more inclined than rural women to use various media, information, and written papers, as well as have more understanding about their health. Another factor might be that you are too worried about other hobbies, your family, and your self-image or you do not have access to enough health care.

Women who were exposed to the media were more likely to use four and more ANC services than women who were not exposed to the media. This finding is similar to studies conducted in Ethiopia [30], Vietnam [31], and Tanzania [35]. Access to the media may have resulted in the more frequent use of ANC visits because the media may have offered relevant health promotion messages, such as the advantages of ANC and information on the danger signs of probable pregnancy problems [35]. Women who had to travel more than two hours to reach the nearest health facility were less likely to use the recommended four ANC visits. This finding is consistent with studies conducted in Ethiopia [32]. These findings revealed that enhancing access to and distribution of health care, especially in rural areas, should be a primary priority [32]. The proportion of women who had the recommended four ANC visits was lowest among women who had four or more births. This is in line with other studies conducted in Nigeria [27].

The occupation of husbands (work in the governmental or nongovernmental sector) had a significant impact on women to use four or more ANC services in the study area. It is known that most women are socioeconomically dependent on male partners who are decision-makers in households, and this influences maternal health care services utilization in Ethiopia [36]. This study contradicts the previous study in Ari Woreda, South Omo Zone, Ethiopia [37], which states that occupation had no significant effect with optimal ANC visit.

The time needed to get to the nearest HF was an important factor for women to use four or more ANC services in this study. This study was in line with the previous study [11, 12], which shows that increased distance to a health facility decreased ANC services. This might be the distance to a health facility or time taken to get to the nearest HF that decreases the chance of getting health services due to less access to the transportation system and low economy for payment for transportation. In addition, the optimal use of antenatal care in this study had a significant impact on road access to HFs. In fact, the cost of transport and the type of transport depend on the availability of the road as it increases optimal use of antenatal care.

This study identified the behavior of health care providers as an important factor in optimal maternal use of antenatal care. This is because health care workers who had good behavior initiate mothers who use ANC visits and may give further explanation about the importance of optimal antenatal care utilization for mothers. The study showed that the number of live children from mothers was significantly related to optimal use of antenatal care. This might be because as the number of children increases, the mothers' want for pregnancy decreases—which leads to a decrease in the use of ANC visits. Similarly, family size in the household reduces the optimal use of the mother's antenatal care in this study. Because the large size of the family leads to a low economic level, an undesired pregnancy leads to low optimal use of maternal prenatal care.

## 5. Conclusion and Recommendations

The purpose of this study was to find out what factors influence optimal maternal antenatal care utilization in the South Gondar Zone, such as socioeconomic, demographic, health, and environmental factors. The result of this study showed that the magnitude of ANC services was 59%. When compared to the national average, this figure was extremely low. The study found that maternal education, maternal occupation, husband's education, husband's occupation, media exposure, time taken to get the nearest health facility, planned pregnancy, wanted pregnancy, health profession preferred by women, source of information about maternal care, media exposure, residence, and road accessibility to the nearest health facility was significantly associated with optimal maternal antenatal care utilization.

Ensuring the continuity of maternal optimal antenatal care utilization in the area, the government's efforts to establish a functional referral system through the distribution of ambulances to districts and the free maternal care policy should be maintained and strengthened as well as expanding educational programs aimed at educating mothers and their husbands on the benefits of a planned pregnancy, wanted pregnancy, and maternal health care and transportation problem and costs require long-term solutions for rural areas.

## Figures and Tables

**Table 1:** Sociodemographic characteristics of respondents in South Gondar Zone.

Variable	Categories	Frequency (%)
Age of respondents	<25	48 (11.1)
	25–29	91 (21.0)
	30–34	107 (24.7)
	>34	188 (43.3)

Respondent religion	Orthodox	390 (89.9)
	Muslim	37 (8.5)
	Others	7 (1.6)

Education level of respondent	Illiterate	194 (44.7)
	Only read and write (no formal education)	35 (8.1)
	Primary educated (grades 1-8)	85 (19.6)
	Secondary school (grades 9-12)	49 (11.3)
	Tertiary level	71 (16.4)

Respondent occupation	Housewife	152 (35.0)
	Farmer	157 (36.2)
	Governmental or nongovernmental employee	62 (14.3)
	Merchant	55 (12.7)
	Daily laborer	8 (1.8)

Education level of husband	Illiterate	155 (35.7)
	Only read and write (no formal education)	57 (13.1)
	Primary educated (grades 1-8)	75 (17.3)
	Secondary school (grades 9-12)	60 (13.8)
	Tertiary level	87 (20.0)

Husband's occupation	Farmer	228 (52.5)
	Governmental or nongovernmental employee	100 (23.0)
	Merchant	81 (18.7)
	Daily laborer	25 (5.8)

Family size	2–4	175 (40.3)
	5–7	188 (43.3)
	8 and above	71 (16.4)

Monthly income	≤1000	63 (14.5)
	1001–2500	124 (28.6)
	2501–5000	177 (40.8)
	5001 and above	70 (16.1)

Owns mobile telephone	No	209 (48.2)
	Yes	225 (51.8)

The decision of maternal care service utilization	Husband	14 (3.2)
	Wife	90 (20.7)
	Both	330 (76.0)

Access to mass media	Exposed to mass media	190 (43.8)
	Not exposed to mass media	244 (56.2)

Residence	Urban	182 (41.9)
	Rural	252 (58.1)

Marital status	Married	390 (89.9)
	Divorced	24 (5.5)
	Windowed	14 (3.2)
	Single	6 (1.4)

**Table 2:** Obstetric characteristics of respondents in the South Gondar Zone.

Variable	Categories	Frequency	Percentage
Birth order	First	81	18.7
	2–3	156	35.9
	4 and above	197	45.4

Counseling about maternal health care	No	90	20.7
	Yes	344	79.3

Size of child at birth	Small	111	25.6
	Average	240	55.3
	Large	83	19.1

Maternal health insurance	No	162	37.3
	Yes	272	62.7

Health provider behavior	Good	364	83.9
	Poor	70	16.1

Postdelivery shelter	No	42	9.7
	Yes	392	90.3

The health facility in kebele	No	61	14.1
	Yes	373	85.9

Nearest health institution	Health center	387	89.2
	Privet clinic	14	3.2
	Hospital	33	7.6

Duty service of maternal health care	No	172	39.6
	Yes	262	60.4

Transportation	On foot	293	67.5
	By car	141	32.5

Number of living children	1–3	241	55.5
	4 and above	193	44.5

Experienced child death	No	325	74.9
	Yes	109	25.1

Road access to the HF	No	235	54.1
	Yes	199	45.9

Place of the previous delivery	Home	187	43.1
	Health institution	247	56.9

Faced any health problem in the last pregnancy	No	279	64.3
	Yes	155	35.7

Planned pregnancy	No	65	15.0
	Yes	369	85.0

Wanted pregnancy	No	65	15.0
	Yes	369	85.0

Experienced death of neonate aged less than seven days	No	366	84.3
	Yes	68	15.7

History of abortion	No	355	81.8
	Yes	79	18.2

Age at first pregnancy	<20	113	26.0
	≥20	321	74.0

Age at first marriage	Less than 15	57	13.1
	15–19	146	33.6
	20–24	205	47.2
	≥25	26	6.0

The time taken to get to the nearest health facility	Below 1 hour	243	56.0
	1–2 hours	162	37.3
	Above 2 hours	29	6.7

**Table 3:** The prevalence of maternal health care in the South Gondar Zone.

Maternal health care	Categories	Frequency	Percentage
ANC	Less than four	178	41.0
	Four and above	256	59.0

**Table 4:** Factors associated with four or more antenatal care (ANC) visits in the South Gondar Zone.

Variable	Four and above ANC		COR (95% CI)	AOR (95% CI)
	No (%)	Yes (%)		
*Education level of respondent*				
Illiterate	124 (69.7)	70 (27.3)	1	1
Only read and write (no formal education)	21 (11.8)	14 (5.5)	1.181 (0.565, 2.468)^∗^	1.282 (0.582, 2.826)^∗^
Primary educated (grade1-8)	10 (5.6)	75 (29.3)	13.286 (6.454, 27.348)^∗^	12.431 (5.739, 26.929)^∗^
Secondary school (grade 9-12)	11 (6.2)	38 (14.8)	6.119 (2.942, 12.727)^∗^	8.205 (3.406,19.767)^∗^
Tertiary level	12 (6.7)	59 (23.0)	8.710 (4.384, 17.301)^∗^	6.406 (2.229,18.416)^∗^
*Education level of husband's*				
Illiterate	91 (51.1)	64 (25.0)	1	1
Only read and write (no formal education)	32 (18.0)	25 (9.8)	1.111 (0.602, 2.051)^∗^	1.080 (0.583, 2.001)^∗^
Primary educated (grades 1-8)	26 (14.6)	49 (19.1)	2.680 (1.511, 4.753)^∗^	2.309 (1.272, 4.193)^∗^
Secondary school (grades 9-12)	10 (5.6)	50 (19.5)	7.109 (3.357, 15.058)^∗^	5.967 (2.753, 12.936)^∗^
Tertiary level	19 (10.7)	68 (26.6)	5.089 (2.791, 9.279)^∗^	4.056 (2.117, 7.769)^∗^
*Husband's occupation*				
Farmer	131 (73.6)	97 (37.9)	1	1
Governmental or nongovernmental employee	22 (12.4)	78 (30.5)	4.788 (2.787,8.226)^∗^	3.736 (2.093, 6.669)^∗^
Merchant	19 (10.7)	62 (24.2)	4.407 (2.475,7.848)^∗^	4.009 (2.235, 7.192)^∗^
Daily laborer	6 (3.4)	19 (7.4)	4.277 (1.646,11.110)^∗^	3.221 (1.202, 8.630)^∗^
*Mass media exposure*				
Exposed to mass media	58 (32.6)	132 (51.6)	1	1
Not exposed to mass media	120 (67.4)	124 (48.4)	0.454 (0.305, 0.676)^∗^	0.520 (0.345, 0.783)^∗^
*Time taken to get the nearest HF*				
Below 1 hour	75 (42.1)	168 (65.6)	1	1
1–2 hours	85 (47.8)	77 (30.1)	0.404 (0.268, 0.610)^∗^	0.494 (0.312, 0.780)^∗^
Above 2 hours	18 (10.1)	11 (4.3)	0.273 (0.123, 0.606)^∗^	0.332 (0.146, 0.757)^∗^
*Planned pregnancy*				
Yes	142 (79.8)	227 (88.7)	1.984 (1.166, 3.379)^∗^	1.912 (1.117, 3.271)^∗^
No	36 (20.2)	29 (11.3)	1	1
*Wanted pregnancy*				
Yes	140 (78.7)	229 (89.5)	2.302 (1.347, 3.936)^∗^	2.341 (1.366, 4.009)^∗^
No	38 (21.3)	27 (10.5)	1	1
*The decision of maternal care service utilization*				
Husband	10 (5.6)	4 (1.6)	1	1
Wife	40 (22.5)	50 (19.5)	3.125 (0.912, 10.711)^∗^	2.516 (0.716, 8.844)^∗^
Both	128 (71.9)	202 (78.9)	3.945 (1.212, 12.846)^∗^	3.517 (1.058, 11.687)^∗^
*Health profession preferred by women*				
Yes	35 (19.7)	98 (38.3)	2.534 (1.620, 3.964)^∗^	2.192 (1.371, 3.507)^∗^
No	143 (80.3)	158 (61.7)	1	1
*Source of information about maternal care*				
Health institution by health care workers	60 (33.7)	164 (64.1)	1	1
Health extension workers	75 (42.1)	52 (20.3)	0.254 (0.160, 0.402)^∗^	0.266 (0.167, 0.425)^∗^
Radio/television	12 (6.7)	24 (9.4)	0.732 (0.344, 1.554)	0.744 (0.347, 1.596)
Relatives and friends	31 (17.4)	16 (6.2)	0.189 (0.096, 0.370)^∗^	0.214 (0.109, 0.424)^∗^
*Owns mobile telephone*				
Yes	54 (30.3)	171 (66.8)	4.620 (3.060, 6.975)^∗^	5.087 (3.286, 7.875)^∗^
No	124 (69.7)	85 (33.2)	1	1
*Residence*				
Urban	40 (22.5)	142 (55.5)	1	1
Rural	138 (77.5)	114 (44.5)	0.233 (0.151, 0.358)^∗^	0.267 (0.164, 0.435)^∗^
*Road access to the HF*				
Yes	60 (33.7)	139 (54.3)	2.336 (1.572, 3.473)^∗^	1.919 (1.270, 2.900)^∗^
No	118 (66.3)	117 (45.7)	1	1
*Health provider behavior*				
Good	133 (74.7)	231 (90.2)	1	1
Poor	45 (25.3)	25 (9.8)	0.320 (0.188, 0.545)^∗^	0.374 (0.213, 0.656)^∗^
*The health facility in kebele*				
Yes	137 (77.0)	236 (92.2)	3.531 (1.988, 6.273)^∗^	3.663 (2.053, 6.538)^∗^
No	41 (23.0)	20 (7.8)	1	1
*Duty service of maternal health care*				
Yes	78 (43.8)	184 (71.9)	3.276 (2.191, 4.899)^∗^	3.101 (2.050, 4.689)^∗^
No	100 (56.2)	72 (28.1)	1	1
*Family size*				
2–4	42 (23.6)	133 (52.0)	1	1
5–7	95 (53.4)	93 (36.3)	0.309 (0.197, 0.485)_∗_	0.297 (0.187, 0.471)^∗^
8 and above	41 (23.0)	30 (11.7)	0.231 (0.129, 0.415)^∗^	0.204 (0.112, 0.375)^∗^
*Number of living children*				
1–3	71 (39.9)	170 (66.4)	1	1
4 and above	107 (60.1)	86 (33.6)	0.336 (0.226, 0.499)^∗^	0.366 (0.236, 0.567)^∗^

1: reference category; ^∗^significance at 5% of the level of significance.

## Data Availability

The data used to support the findings of this study are available from the corresponding author upon request.

## Abbreviations

ANC: Antenatal care
AOR: Adjusted odds ratio
CI: Confidence interval
COR: Crude odds ratio
DHS: Demographic and Health Survey
HF: Health facility
SPSS: Statistical Package for Social Science
WHO: World Health Organization.
